# Evaluation of antidepressant-like effects of aqueous and ethanolic extracts of *Pimpinella anisum* fruit in mice

**Published:** 2016

**Authors:** Zahra Shahamat, Saeid Abbasi-Maleki, Saeid Mohammadi Motamed

**Affiliations:** 1 *Depatment of Pharmacology, Pharmaceutical Sciences Branch, Islamic Azad University, Tehran, Iran*; 2 *Department of Pharmacology, Urmia Branch, Islamic Azad University, Urmia, Iran*; 3 *Department of Pharmacognosy, Pharmaceutical Science Branch, Islamic Azad University, Tehran, Iran*

**Keywords:** *Pimpinella anisum*, *Forced swimming test*, *Tail suspension test*, *Antidepressant activity*, *Mice*

## Abstract

**Objective:**

*Pimpinella anisum *(*P. anisum****)*** has different pharmacological properties such as anticonvulsant, analgesic, tranquilizer, antidepressant and anti-anxiety effects. In this study the antidepressant-like effect of aqueous and ethanolic extracts of P.* anisum* fruit in mice was investigated.

**Materials and Methods:**

Forced swimming test (FST) and tail suspension test (TST) were used to determine the antidepressant effects of *P. anisum *(50, 100 and 200 mg/kg, i.p.) fruit extracts. Fluoxetine (20 mg/kg, i.p.) and imipramine (30 mg/kg, i.p.) were used as standard drugs.

**Results:**

All the three doses of aqueous and ethanolic extracts (except 50 mg/kg of aqueous extract in FST) significantly and dose-dependently reduced the immobility times in both FST and TST. All doses of extracts increased the swimming time dose-dependently, without any significant change in climbing time. In addition, all doses of ethanolic extract reduced immobility times and increased swimming time insignificantly higher than aqueous extract. But, the two extracts decreased the duration of climbing time similarly. Fluoxetine and imipramine decreased immobility time in both tests. Fluoxetine increased the swimming time without modifying climbing time. In contrast, imipramine increased climbing time without any significant change in swimming time.

**Conclusion:**

The results of this study suggest that *P*.* anisum* possesses an antidepressant-like activity similar to that of fluoxetine, which has a potential clinical value for application in the management of depression.

## Introduction

Depression is a serious mood disorder that interferes with an individual’s thoughts, behaviour, feelings and enjoying life. Today, depression is estimated to affect more than 350 million individuals worldwide (Kessler and Ustun, 2008). It has been revealed that symptoms of depression are associated with decreased levels of monoaminergic transmitters such as noradrenaline, 5-hydroxytriptamine (5-HT) and dopamine in the brain (Meyers, 2000). The main used antidepressant agents are tricyclic antidepressants (TCAs) and selective serotonin reuptake inhibitors (SSRIs), but these drugs have significant adverse effects in long period administration. Herbal antidepressants are increasingly being introduced to treat severe depression and many of these agents are reported to have a rate of efficacy comparable to the medications with fewer side effects (Woode et al., 2010).

*Pimpinella anisum *L. (*P*. *anisum*) is a plant belonging to the Umbelliferae family. It is an annual grassy herb, 30-50 cm high with white flowers and small green/yellow seeds, and native to Mediterranean countries (Salehi Surmaghi, 2010). Anise fruits (seeds), also known as aniseed, contain 1.5 - 5.0% essential oil with anethole, a phenylpropanoid, as major component. Moreover, aniseed essential oil contains small quantity of estragol, anisaldehyde, γ-himachalene and *cis*-anethole (Gulcin et al., 2003; Rodriguez et al., 2003; Salehi Surmaghi, 2010; Zargari, 2011). Aniseeds in Iranian traditional medicine are used as disinfectant, carminative, diuretic, aromatic, analgesic, increasing agent of milk production, and tranquilizer, for menstruation, hepatoprotective effect, relief of the nightmares, dysmenorrheal and menopausal hot flashes in women as well as to treat epilepsy and seizure (Salehi Surmaghi, 2010; Zargari, 2011; Shoji and Abdollahi Fard, 2012). In addition, different studies showed beneficial effects of *P. anisum* on memory disorder, depression, cerebral ischemia, anxiety and Alzheimer disease (De Sousa et al., 2011; EL-Hodairy, 2014; Niksokhan et al., 2015). Given this background, the aim of this study is to investigate the possible antidepressant effects of aqueous and ethanolic extracts of *P. anisum *fruit in mice.

## Materials and Methods

**Plant material and preparation of extracts**

Fruits of *P*. *anisum *were obtained from a local market in Tehran, Iran. Herbarium of Tehran University and voucher samples were kept for reference at the herbarium in Department of Pharmacognosy, School of Pharmacy, Tehran, Iran (Voucher No. 723.2 TEH).

The fruits were dried in shadow and pulverized using a grinder-mixer. Then, 50 g dried fruits powder was macerated separately for 48h in 200 ml water and 70% (v/v) ethanol for aqueous and ethanolic extraction, respectively. The solvents of extracts were removed at room temperature to be dried, and the dried extracts were kept in clean vials in cool conditions.

**Drugs**

The purified powder of imipramine hydrochloride (Pars Daru, Tehran, Iran) and fluoxetine hydrochloride (Arya Pharmaceutical Co, Tehran, Iran) were used in this study.

**Animals**

Male Naval Medical Research Institute (NMRI) mice (weighing 20-30 g), from Pasteur Institute (Tehran, Iran), were used for tests. The animals were maintained at 22*±*1*°*C with free access to water and food, under a 12:12 h light/dark cycle (lights on at 07:00 A.M.). All manipulations were carried out between 8:00 A.M. and 3:00 P.M., and each animal was used only once. All procedures were performed per the guidelines approved by School of Medicine, Tehran University of Medical Science. 

**Experimental design and animal groups**

In the present study, all mice were randomly divided into 18 different groups. Each group consisted of six mice according to forced swimming test (FST) and trail suspension test (TST):

Groups 1 and 2: Normal saline 10ml/kg; as control for FST and TST.

Groups 3 and 4: Fluoxetine 20 mg/kg; as standard drug for FST and TST.

Groups 5 and 6: Imipramine 30 mg/kg; as standard drug control for FST and TST.

Groups 7, 8 and 9: Three different doses of aqueous extract of *P. anisum (*50, 100 and 200 mg/kg) for FST.

Groups 10, 11 and 12: Three different doses of aqueous extract of *P.anisum (*50, 100 and 200 mg/kg) for TST.

Groups 13, 14 and 15: Three different doses of ethanolic extract of *P.anisum (*50, 100 and 200 mg/kg) for FST.

Groups 16, 17 and 18: Three different doses of ethanolic extract of *P. anisum (*50, 100 and 200 mg/kg) for TST.

The doses of the drugs and extracts used were selected according to previous studies (Heidari and Ayeli, 2005; Moallem et al., 2007).

In this study, all drugs and extracts were dissolved in normal saline (0.9%) and administered intraperitoneally (i.p.) at a constant volume of 10 ml/kg.At 30 min after single administration of drugs and extracts, all FST and TST observations were made.

**FST**

The FST was carried out on mice individually. They were forced to swim in an open cylindrical container (10 cm diameter × 25 cm height), filled with 15 cm of water at 25±1°C (Porsolt et al., 1977). In this test the total duration of immobility, climbing and swimming behaviors was recorded for the last 4 min within a 6-min test using a chronometer (Zomkowski et al., 2005). In this method, decrease in immobility time and increases in climbing or swimming time were considered as behavioral responses to antidepressant-like activity (Zomkowski et al., 2005).

**TST**

In this test, mice were isolated from exposure to sound and vision and suspended 50 cm above the floor by adhesive tape placed about 2 cm from the tip of the tail. In TST, immobility time was recorded for the last 4 min within a 6-min test. The duration of immobility was recorded using a chronometer (Steru et al., 1985).

**Statistical analysis**

Data were analyzed using SPSS version 17.0. All the data are expressed as mean ± SEM. The data were analyzed by one-way analysis of variance (ANOVA), followed by Tukey's post-hoc test to determine the statistical significance between groups. p < 0.05 was considered as the level of significance.

## Results

**Effects of aqueous and ethanolic extracts of** ***P. anisum*** **on immobility time in FST**

**Figure 1:**
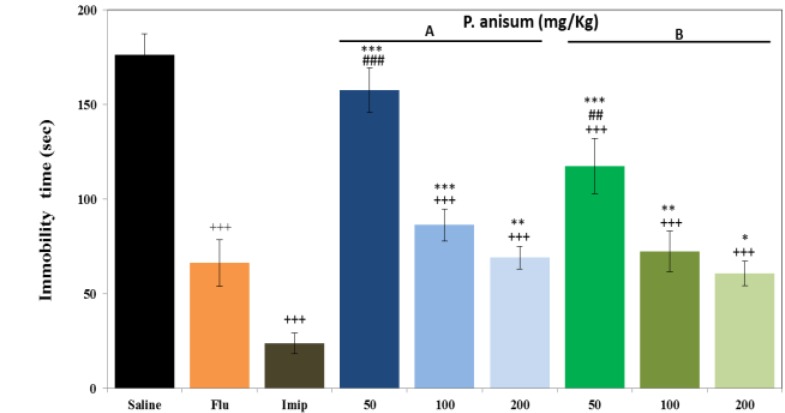
Effects of *P. anisum* (50, 100 and 200 mg/kg) aqueous (Panel A) and ethanolic extracts (Panel B), fluoxetine (Flu, 20 mg/kg) and imipramine (Imip, 30 mg/kg) on duration of immobility time in FST. Data are presented as mean ± SD (n=6). +++ p<0.001 compared with saline-treated group. ##p<0.01 and ### p<0.001 compared with fluoxetine- treated group. *p<0.05, **p<0.01 and ***p<0.001 compared with imipramine- treated group.

As illustrated in Figure 1A and B, only100 and 200 mg/kg of aqueous extract (50.96 and 60.78 %, respectively, p<0.001 for both cases) and all three doses of ethanolic extract (42.57, 58.92 and 65.53 %, respectively, p<0.001 for all cases) reduced the immobility time in FST compared to the control group in a dose-dependent manner. In addition, all doses of ethanolic extract insignificantly higher than aqueous extract reduced immobility time. Fluoxetine (20mg/kg) and imipramine (30mg/kg) decreased immobility time (62.42% and 86.52%, respectively, p<0.001 for both cases) as compared to control group (Figure 1). The results relieved that only high dose (200 mg/kg) of ethanolic extract reduced immobility time insignificantly higher than fluoxetine. But, fluoxetine higher than low dose (50mg/kg) of aqueous and ethanolic extracts reduced immobility time (p<0.001 and p<0.01, respectively). The results also showed the effect of that all doses of aqueous (p<0.001, p<0.01 and p<0.01, respectively) and ethanolic extracts (p<0.001, p<0.01 and p<0.05, respectively) on reduction of immobility time were lower than imipramine.

**Effects of aqueous and ethanolic extracts of** ***P. anisum*** **on swimming time in FST**

**Figure 2:**
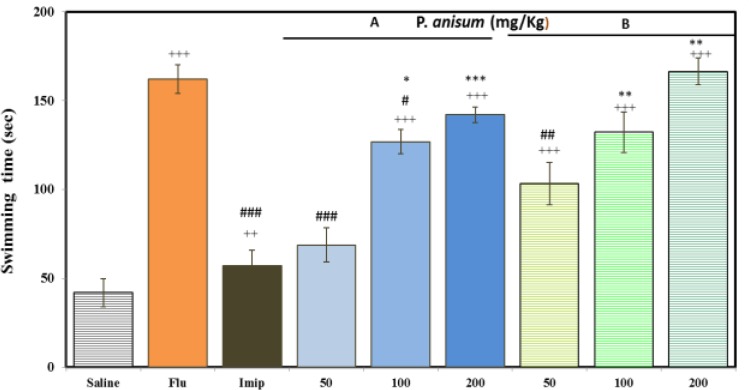
Effects of *P. anisum* (50, 100 and 200 mg/kg) aqueous (Panel A) and ethanolic extracts (Panel B), fluoxetine (Flu, 20 mg/kg) and imipramine (Imip, 30 mg/kg) on duration of swimming time in FST. Data are presented as mean ± SD (n=6). +++ p<0.001 compared with saline-treated group. #p<0.05, ##p<0.01 and ### p<0.001 compared with fluoxetine-treated group. *p<0.05, **p<0.01 and ***p<0.001 compared with imipramine-treated group

Figure 2A and B illustrates that only 100 and 200 mg/kg of aqueous (202.53 and 238.54 %, respectively, p<0.001 for both cases) and all three doses of ethanolic extract (170.00, 215.42 and 296.88%, respectively, p<0.001 for all cases) significantly increased the swimming time compared to the control group and in a dose-dependent manner. Similar to immobility time, swimming time also increased insignificantly by ethanolic extract compared to aqueous extract. Fluoxetine, unlike imipramine, increased swimming time (286.56%, p<0.001). Immobility time, only due to high dose (200mg/kg) of ethanolic extract increased swimming time insignificantly higher than fluoxetine (p>0.05). However, Fluoxetine increased the swimming time higher than 50 and 100 mg/kg of aqueous extract (p<0.001 and p<0.05, respectively) and low dose (50mg/kg) of ethanolic extract (p<0.01). In contrast, the effect of imipramine on increased the swimming time was lower than 100 and 200 mg/kg of aqueous (p<0.05 and p<0.01, respectively) and ethanolic extracts (p<0.01 and p<0.001, respectively) (Figure 2).

**Effects of aqueous and ethanolic extracts of** ***P. anisum*** **on climbing time in FST**

**Figure 3:**
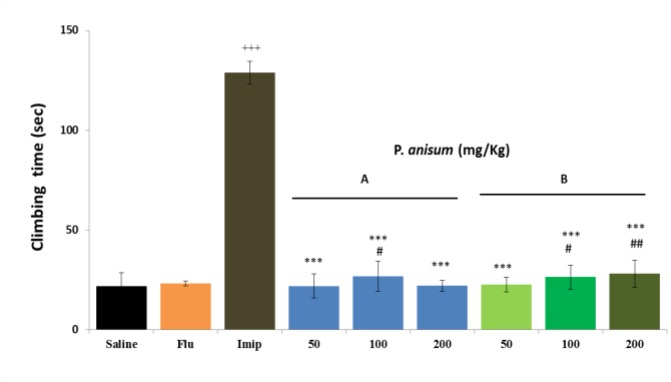
Effects of *P. anisum* (50, 100 and 200 mg/kg) aqueous (Panel A) and ethanolic extracts (Panel B), fluoxetine (Flu, 20 mg/kg) and imipramine (Imip, 30 mg/kg) on duration of climbing time in FST. Data are presented as mean ± SD (n=6). +++ p<0.001 compared with saline-treated group. ***p<0.001 compared with imipramine- treated group

Figure 3A and B illustrates that climbing time was not significantly increased by both aqueous (0.50, 22.49 and 0.73%, respectively) and ethanolic extract (4.27, 21.02 and 28.77%, respectively) and fluoxetine (6.01%). In contrast, imipramine significantly increased climbing time (491.23%, p<0.001) compared to control group (Figure 3). Both extracts decreased the duration of climbing time simillarly. The results showed that only middle dose (100 mg/kg) of aqueous extract and 100 and 200 mg/kg of ethanolic extract increased climbing time insignificantly higher than fluoxetine. However, the effect of imipramine on increased climbing time was higher than all three doses of both extracts (p<0.001 for all cases). 

**Effects of aqueous and ethanolic extracts of** ***P. anisum*** **on immobility time in TST**

**Figure 4:**
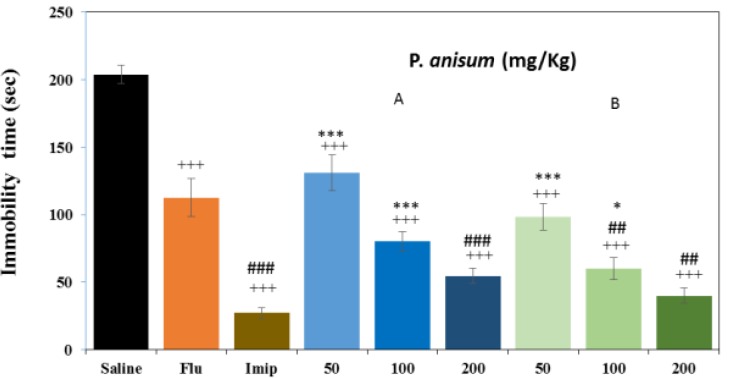
Effects of *P. anisum* (50, 100 and 200 mg/kg) aqueous (Panel A) and ethanolic extracts (Panel B), fluoxetine (Flu, 20 mg/kg) and imipramine (Imip, 30 mg/kg) on duration of immobility time in TST. Data are presented as mean ± SD (n=6). +++ p<0.001 compared with saline-treated group. ##p<0.01 and ### p<0.001 compared with fluoxetine- treated group. *p<0.05 and***p<0.001 compared with imipramine- treated group

As depicted in Figure 4A and B, all three doses (50,100 and 200 mg/kg) of aqueous (26.89, 60.73 and 73.23%, respectively, p<0.001 for all cases) and ethanolic extracts (52.65, 70.62 and 80.42%, respectively, p<0.001 for all cases) reduced the immobility time compared to the control group significantly and in a dose-dependent manner. Similar to FST, all doses of ethanolic extract insignificantly higher than aqueous extract reduced immobility time in TST. Fluoxetine (20mg/kg) and imipramine (30mg/kg) also significantly reduced the immobility time compared to the control group (44.80% and, 86.73%, respectively, p<0.001 for both cases) (Figure 4). Only the effects of 200 mg/kg of aqueous extract (p<0.001) and 100 and 200 mg/kg of ethanolic extract (p<0.01 for both cases) reduced immobility time higher than fluoxetine. However, the effect of imipramine on immobility time reduction was higher than 50 and 100 mg/kg of aqueous (p<0.001, for both cases) and ethanolic extracts (p<0.001 and p<0.05, respectively).

## Discussion

The present study revealed that different doses of aqueous and ethanolic extracts of* P. anisum* fruit showed significant antidepressant-like activity by both FST and TST. Both of these tests are commonly used as the standard models of depression in animals. These tests are quite sensitive and widely employed to study the rodent behaviors, particularly mice, to predict the antidepressant potential determined by a decrease in immobility time (Porsolt et al., 1977). It has been reported that TST is less stressful and has higher pharmacological sensitivity than FST (Thierry et al., 1986). Different studies have shown that swimming is sensitive to serotonergic agents such as fluoxetine (a serotonin reuptake inhibitor), and climbing is sensitive to TCAs and drugs with selective effects on noradrenergic transmission (Detke et al., 1995; Page et al., 1999). Our results are in agreement with other reports demonstrating that fluoxetine reduces the immobility time and increases the swimming time, but do not affect the climbing time. 

However, imipramine increased the climbing time without any significant change in swimming time (Detke et al., 1995; Emamghoreishi and Talebianpour, 2009). All doses of aqueous and ethanolic extracts of *P. anisum *reduced immobility time and increased swimming time in a dose-dependent manner without significantly affecting the climbing time by FST. Our results demonstrated that only high dose (200 mg/kg) of ethanolic extract insignificantly reduced immobility time higher than fluoxetine in FST. Unlike FST, only high dose (200 mg/kg) of aqueous extract and 100 and 200 mg /kg of ethanolic extract significantly reduced immobility time higher than fluoxetine in TST. However, all three doses of aqueous and ethanolic extracts, less pronouncedly than imipramine (30mg/kg), affect immobility and climbing behaviors.

 Also, only high dose (200 mg/kg) of ethanolic extract increased swimming time insignificantly higher than fluoxetine, but all doses of extracts increased swimming time more pronouncedly than imipramine. Therefore, according to the results of these tests, the behavior pattern that is induced by the extracts by the FST is similar to that of fluoxetine (decrease in immobility time, increase in swimming time), and it seems serotonergic neurotransmission has a key role in antidepressant-like activity of *P. anisum* extracts. Nevertheless, the pharmacological mechanism and the compound responsible for antidepressant-like activity of* P. anisum *could not be identified in the present study.

Several investigations revealed that* P. anisum *fruit consists of several active compounds and all of them may be responsible for the antidepressant-like effects. Previous studies have shown that this plant contains anethole, estragole, anisaldehyde, γ-himachalene and *cis*-anethole (Gulcin et al., 2003; Rodriguez et al., 2003). However, the major compound of *P. anisum *is anethole, responsible for the medicinal properties (Salehi Surmaghi, 2010; Zargari, 2011; Shojaii and Abdollahi Fard, 2013; EL-Hodairy, 2014). Anethole is an antioxidant that is slightly soluble in water but shows high solubility in ethanol. Hence, stronger antidepressant-like effects of ethanolic extract compared to the aqueous extract is probably due to the higher concentration of anethole in the ethanolic extract. Previous studies have demonstrated antidepressant-like activity of antioxidants (Scapagnini et al., 2012). On the other hand, different studies have demonstrated that antioxidants could inhibit the reuptake of 5-HT (Weinstock et al., 2002; Khanzode et al., 2003). Moreover, there is a report on inhibition of monoamine-oxidase (MAO) by anethole (Drukarch et al., 2006). Hence, inhibition of 5-HT reuptake as well as inhibition of MAO by anethole could increase 5-HT availability in synaptic clefts, which could desensitize the 5-HT receptors involved in depression.

In conclusion, the results of this study revealed that *P. anisum* may have potential therapeutic application for the management of depressive disorders, and this effect is comparable to fluoxetine. However, further mechanistic studies are required to elucidate the exact mechanism of antidepressant-like activity of *P. anisum *fruit extracts.
